# Literature-Grounded Simulation of Non-Invasive Glucose Monitoring Using NIR Wearable Sensor Archetypes: Accuracy Metrics, Candidate Confounders, and an Adaptive Calibration Framework

**DOI:** 10.3390/s26175443

**Published:** 2026-08-28

**Authors:** David Alberto García-Arango, José Alexander Velásquez Ochoa, Luis Fernando Garcés Giraldo, Natalia Isabel Jaramillo Gómez

**Affiliations:** 1 Research Directorate, Universidad Autónoma del Perú, Villa EL Salvador, Lima 15042, Peru; 2 Research Departament, Tecnológico de Antioquia, Medellín 050036, Colombia; jose.velasquez46@tdea.edu.co (J.A.V.O.); natalia.jaramillo24@tdea.edu.co (N.I.J.G.); 3 Posgraduate School, Universidad Continental, Av. Alfredo Mendiola 5210, Lima 15306, Peru

**Keywords:** NIR spectroscopy, glucose monitoring, wearable sensors, simulation, adaptive calibration

## Abstract

**Highlights:**

**What are the main findings?**

- A literature-grounded synthetic dataset of 320 glucose pairs yielded a mean MARD of 10.38%, a reference–estimate correlation of r = 0.958, and 89.4% of records assigned to Clarke Zone A; these values characterize the simulation and are not clinical-validation results.
- Correlations between MARD and eight candidate physiological/environmental factors were small (|r| ≤ 0.090) and none was statistically supported after Holm correction; the sensor-archetype MARD distributions also did not differ significantly.

**What are the implications of the main findings?**

- The results support a transparent, literature-grounded methodological framework for evaluating calibration hypotheses, rather than establishing clinical or regulatory performance of a wearable device.
- Prospective work should use participant-linked raw optical spectra, grouped subject-level validation, documented measurement geometry, skin-phototype characterization, and prospectively implemented adaptive calibration in wearable hardware.

**Abstract:**

Non-invasive continuous glucose monitoring remains an important technological challenge in diabetes management. This study was revised as a literature-grounded simulation and methodological proof-of-concept rather than an experimental clinical validation. The analysis used 320 independent synthetic reference–NIR glucose pairs generated from physiological ranges and performance distributions reported in the cited literature, together with an illustrative 576-point, 48-h synthetic time series. Three sensor archetypes were informed by published optical systems. Descriptive agreement was evaluated using mean absolute relative difference (MARD), Pearson correlation, Bland–Altman analysis, and the supplied Clarke Error Grid (CEG) categories. Candidate physiological and environmental factors were examined using Pearson correlations with 95% confidence intervals, raw *p*-values, and Holm correction. The synthetic dataset produced an overall MARD of 10.38% (SD = 7.67%), r = 0.958 (R^2^ = 0.918, *p* < 0.001), a Bland–Altman bias of 4.32 mg/dL, and 89.4%/10.3%/0.3% of records in CEG zones A/B/D, respectively. All candidate-factor correlations were small and non-significant after multiplicity correction; the largest was hematocrit (r = 0.090, 95% CI −0.019 to 0.198; Holm-adjusted *p* = 0.852). Differences in MARD among sensor archetypes were also non-significant (ANOVA *p* = 0.369; Kruskal–Wallis *p* = 0.543). Adaptive calibration is therefore presented as a literature-informed framework for future prospective validation, not as a performance improvement demonstrated by the present synthetic dataset. No FDA or ISO compliance or clinical applicability is claimed.

## 1. Introduction

Non-invasive continuous glucose monitoring is a longstanding objective in diabetes technology because it could reduce the burden associated with repeated finger-stick sampling and subcutaneous sensor insertion [1,2]. Optical approaches are attractive because they can interrogate tissue without direct access to blood, but clinical translation remains difficult because the glucose-related optical signal is weak relative to background absorption and scattering from water, hemoglobin, lipids, melanin, and other tissue constituents [1,2,3,4]. Consequently, reported performance depends strongly on the optical configuration, measurement site, calibration strategy, and validation design.

Near-infrared (NIR) glucose sensing exploits changes in tissue attenuation associated with overtone and combination vibrations of C–H and O–H bonds. In a diffuse-reflectance or reflected-light configuration, the light source and detector are positioned on the same side of the tissue, and the detector records photons that have undergone absorption and multiple scattering; in transmission or differential-absorbance configurations, source–detector geometry is arranged to quantify wavelength-dependent attenuation through the interrogated tissue. Published wearable prototypes relevant to the present simulation include reflected-light finger/wrist systems using a 940 nm emitter with a 900–1700 nm detector [5], a 940 nm NIR prototype coupled to PSO-ANN modeling [6], and a fully optical visible–NIR differential-absorbance plus photoplethysmography (PPG) system using multimodal temporal modeling [7].

Physiological and environmental variables may influence optical glucose estimation by modifying absorption, scattering, tissue perfusion, sensor–skin coupling, or motion contamination. Candidate variables represented in the supplementary simulation include skin thickness, skin temperature, relative humidity, body mass index (BMI), age, hematocrit, motion artifact, and sensor contact pressure. Skin pigmentation is particularly important for transdermal optical sensing because melanin changes wavelength-dependent attenuation and may affect model transferability [5]. However, Fitzpatrick skin type or an objective melanin index was not encoded in the synthetic dataset; this is treated as a major limitation rather than inferred from the available variables.

The dataset analyzed here is not a set of primary measurements and is not a pooled participant-level extraction from the cited studies. It is a literature-grounded synthetic dataset in which 320 independent reference–NIR glucose pairs were generated from physiological ranges, benchmark error distributions, and device characteristics documented in the source-traceability fields of the Supplementary Workbook. The cited studies therefore provide parameterization and contextual benchmarks, not individual observations [8,9,10,11,12,13,14,15,16,17,18,19]. A separate 576-point, 48-h time series is also synthetic and is used only to illustrate temporal behavior.

Accordingly, the objective of this study is to characterize the internal accuracy metrics of the literature-grounded simulation, quantify the statistical uncertainty associated with candidate confounder–MARD relationships, compare three optical sensor archetypes descriptively, and formulate an adaptive-calibration framework for future prospective wearable implementation. The analysis is explicitly hypothesis-generating and does not test the clinical performance of an actual device.

Regulatory and clinical claims require evidence beyond a single aggregate MARD value. Current U.S. iCGM special controls require robust clinical data across the intended population and measurement range, concentration-specific accuracy calculations with confidence bounds, sensor-wear performance, interference testing, usability evidence, and other controls [20]. ISO 15197:2013 addresses capillary blood-glucose monitoring systems for self-testing by lay users and is not treated here as a compliance standard for the simulated continuous optical system [21]. FDA-recognized CLSI POCT05 provides a more directly relevant framework for performance metrics of continuous interstitial glucose monitoring [22]. These standards are discussed only to delimit interpretation; no regulatory compliance is asserted.

## 2. Materials and Methods

This work is a literature-grounded simulation and methodological analysis. No participants were recruited, no venous or capillary specimens were collected by the authors, and no NIR spectra were acquired in the present study. The Supplementary Workbook (Supplementary File S1, see Appendix A for dataset structure and source traceability) contains 320 synthetic paired records. Each row includes a synthetic reference glucose value, a synthetic NIR-estimated value, MARD, a supplied Clarke Error Grid category, eight candidate physiological/environmental variables, a sensor-archetype label, source identifiers, URLs, and a data-generation note. The rows do not correspond to identifiable participants from the cited studies, and no participant identifier is available. For the analyses reported here, rows are therefore treated as independent synthetic observations; participant-level generalization cannot be assessed. The Supplementary Workbook (Supplementary File S1, see Appendix A for dataset structure and source traceability) contains 320 synthetic paired records.

The synthetic values were generated from literature-reported physiological ranges and performance distributions. The three sensor labels represent literature-informed archetypes. Sensor A is informed by the niGLUC-2.0v reflected-light finger/wrist prototype reported by Rajeswari and Vijayakumar [5], which used a 940 nm emitter and 900–1700 nm detector. Sensor B is informed by the 940 nm NIR/PSO-ANN prototype described by Raghavan et al. [6]. Sensor C is corrected here to represent the fully optical visible–NIR differential-absorbance + PPG multimodal architecture reported by Cheng et al. [7]; it does not represent electrochemical CGM input. The anatomical site, optical configuration, and wavelength information available from the sources are summarized in Table 1. Integration time and repeated-measurement counts were not consistently harmonized in the supplementary dataset and are therefore not imputed or treated as controlled factors.

**Figure 1:**
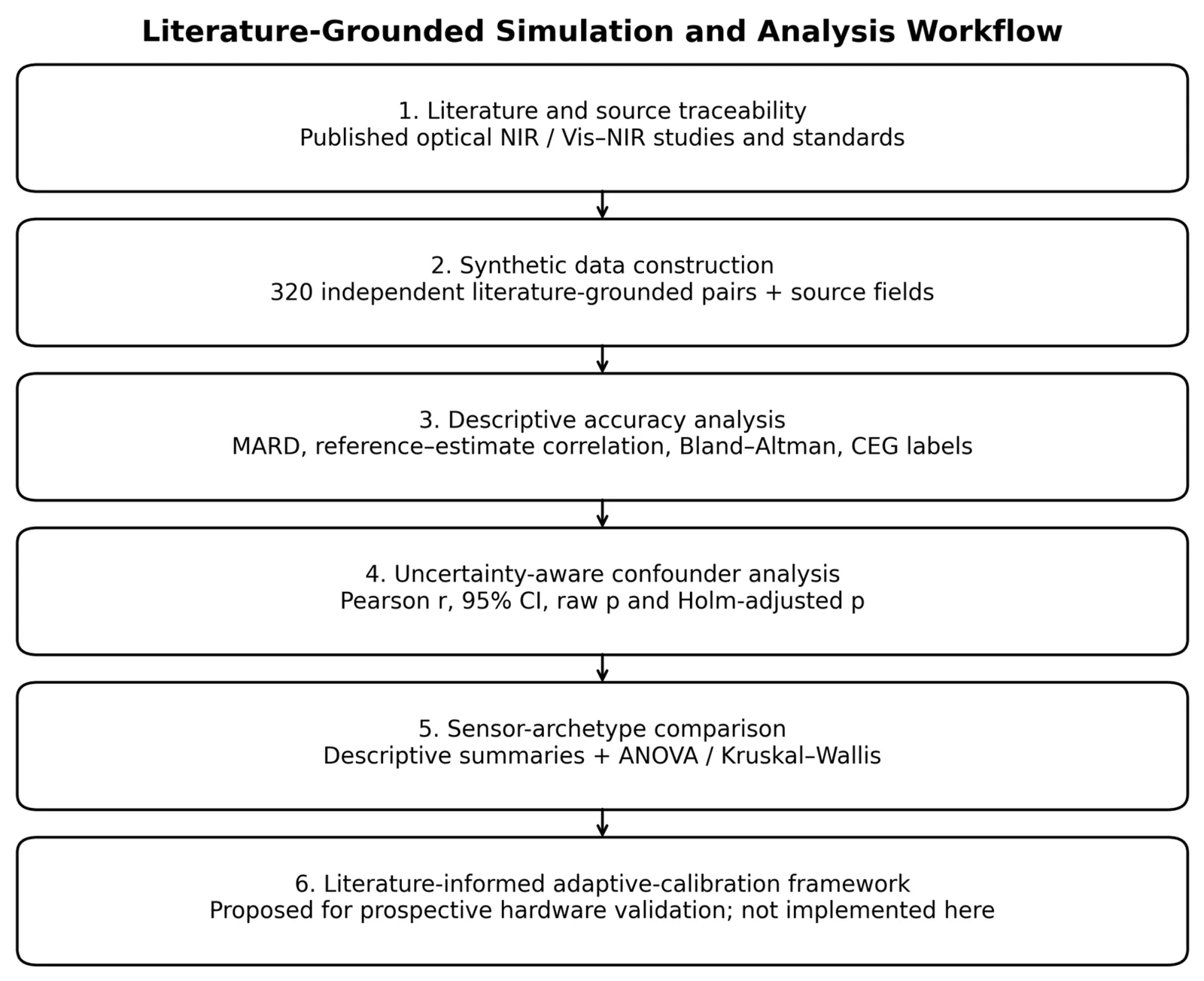
Literature-grounded simulation and analysis workflow. Source studies and technical references were used to establish parameter ranges and sensor-archetype characteristics; 320 synthetic paired records and a separate illustrative 48-h synthetic series were then analyzed. The adaptive-calibration component is a proposed framework for future prospective implementation and was not executed on participant-linked raw spectra in the present study.

The three sensor archetypes described in Table 1 are based on research prototypes and optical configurations reported in the cited source studies and were not commercially purchased, assembled, or experimentally operated by the authors in the present study. Accordingly, no commercial NIR sensor, glucose analyzer, reagent, biological material, or other experimental instrument was used to generate the synthetic dataset. Manufacturer information is therefore not applicable to the present simulation, except where commercial components are explicitly described as part of the referenced source studies. The methodological workflow is shown in Figure 1. 

The principal descriptive outcomes were MARD, reference–estimate Pearson correlation, Bland–Altman bias and 95% limits of agreement, and the CEG category supplied with each synthetic pair. For each candidate physiological/environmental variable, the association with MARD was quantified using Pearson’s r, a Fisher-z 95% confidence interval, a two-sided *p*-value, and Holm adjustment across eight simultaneous tests. Because the simulated sensor groups contained different numbers of records, differences in MARD among archetypes were examined using both one-way ANOVA and the Kruskal–Wallis test. Statistical significance was evaluated at α = 0.05; effect magnitude and confidence intervals were emphasized rather than significance alone.

All statistical analyses were performed using Python version 3.13.5 (Python Software Foundation, Beaverton, OR, USA), with NumPy version 2.3.5 and SciPy version 1.17.0 for numerical and statistical computations. Graphical outputs used for the analytical assessment were generated using Matplotlib version 3.10.8.

No structural equation model, panel regression, leave-one-out cross-validation (LOOCV), or 10-fold cross-validation was performed in the revised analysis because the supplementary data contain neither participant identifiers nor raw spectra required for defensible participant-grouped model validation. In a future prospective dataset with repeated measurements, validation should be grouped at the participant level (for example, leave-one-subject-out or grouped k-fold validation) to prevent measurements from the same individual from appearing in both training and test partitions. Likewise, partial least-squares regression (PLSR), preprocessing, and adaptive model updating should be evaluated on raw spectral data [9,22,23]. The constant-analyte updating strategy described by Gessell and Small [23] is used here only as the conceptual basis for the proposed adaptive-calibration framework. Their approximately 50% improvement was reported for updated PLS models in an in vitro aqueous-sample NIR experiment and is not a result reproduced by the current synthetic dataset.

## 3. Results

Across the 320 synthetic pairs, mean MARD was 10.38% (SD = 7.67%). The synthetic reference and NIR-estimated glucose values averaged 127.7 and 132.0 mg/dL, respectively. The three sensor-archetype groups contained 113, 131, and 76 records for Sensors A, B, and C. Because these records were generated from literature-informed distributions, the following values describe the internal behavior of the simulation and should not be interpreted as observed device accuracy.

The reference–estimate correlation was r = 0.958 (R^2^ = 0.918, *p* < 0.001), and Bland–Altman analysis yielded a mean difference (NIR − reference) of 4.32 mg/dL with 95% limits of agreement from −27.86 to 36.50 mg/dL. Table 2 summarizes the synthetic performance by glucose range and sensor archetype. The distribution of records differed from the earlier manuscript version; the corrected counts and descriptive statistics below are calculated directly from the submitted Supplementary Workbook.

The largest mean MARD among the four glucose-range strata occurred in the hypoglycemic subset (<70 mg/dL; *n* = 53, MARD = 11.45%), whereas the >200 mg/dL subset showed a lower mean MARD of 8.11% in this simulation. Because the records were generated rather than prospectively observed, these range differences should be treated as properties of the simulation and not as evidence that a physical NIR device performs better or worse in a particular glycemic range.

Sensor C had the lowest descriptive mean MARD (9.34%), followed by Sensor A (10.48%) and Sensor B (10.90%); however, these distributions did not differ significantly by one-way ANOVA (F = 0.999, *p* = 0.369) or Kruskal–Wallis testing (H = 1.222, *p* = 0.543). Therefore, the revised manuscript does not rank Sensor C as superior or attribute its descriptive value to a specific architecture. Sensor C is also explicitly defined as a fully optical visible–NIR + PPG archetype, not an electrochemical CGM–NIR fusion device.

**Figure 2:**
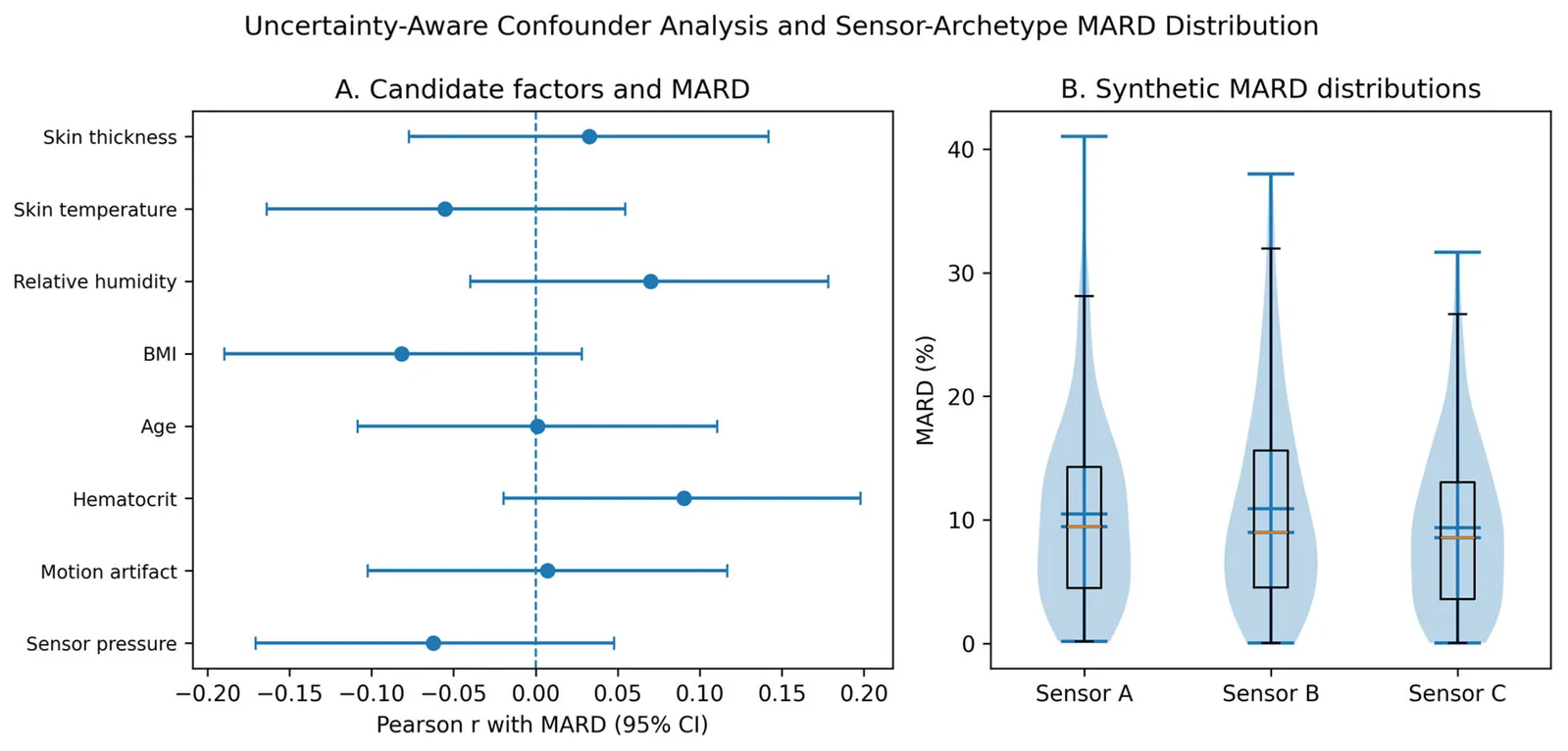
Uncertainty-aware analysis of candidate factors and MARD distributions. Panel (**A**) shows Pearson correlations between MARD and eight candidate variables with 95% confidence intervals; all intervals cross zero. Panel (**B**) shows violin distributions of MARD by sensor archetype with overlaid box plots. The blue horizontal segment within each violin indicates the mean MARD, whereas the orange horizontal segment indicates the median. The black box spans the first to third quartiles (Q1–Q3), and the black whiskers represent the non-outlying range according to the boxplot convention. The blue vertical line and end caps indicate the observed minimum-to-maximum range of the corresponding distribution. No regulatory threshold is superimposed because the dataset is synthetic and no universal FDA MARD cutoff is asserted.

Figure 2 displays the uncertainty of the candidate-factor correlations with MARD and the sensor-archetype MARD distributions. All eight 95% confidence intervals crossed zero, emphasizing that the small observed coefficients do not provide statistical support for individual confounding effects in this synthetic dataset.

The largest positive coefficient was observed for hematocrit (r = 0.090, 95% CI −0.019 to 0.198, *p* = 0.107; Holm-adjusted *p* = 0.852). Relative humidity (r = 0.070), skin temperature (r = −0.055), sensor pressure (r = −0.062), and the other candidate variables likewise showed small coefficients with non-significant adjusted *p*-values. Table 3 reports the complete inferential results. These findings do not identify any variable as a statistically established confounder; instead, the variables remain candidate covariates motivated by optical and physiological literature and should be tested with real participant-linked measurements.

The absence of statistically supported associations does not imply that these variables are unimportant in real optical sensing. Rather, it indicates that the present synthetic parameterization does not contain sufficient independent information to estimate their real-world effects. Skin pigmentation cannot be tested at all because Fitzpatrick phototype and melanin index are absent from the dataset; future experiments should collect and report these measures explicitly, together with anatomical site, contact pressure, ambient conditions, and motion quality indicators.

**Figure 3:**
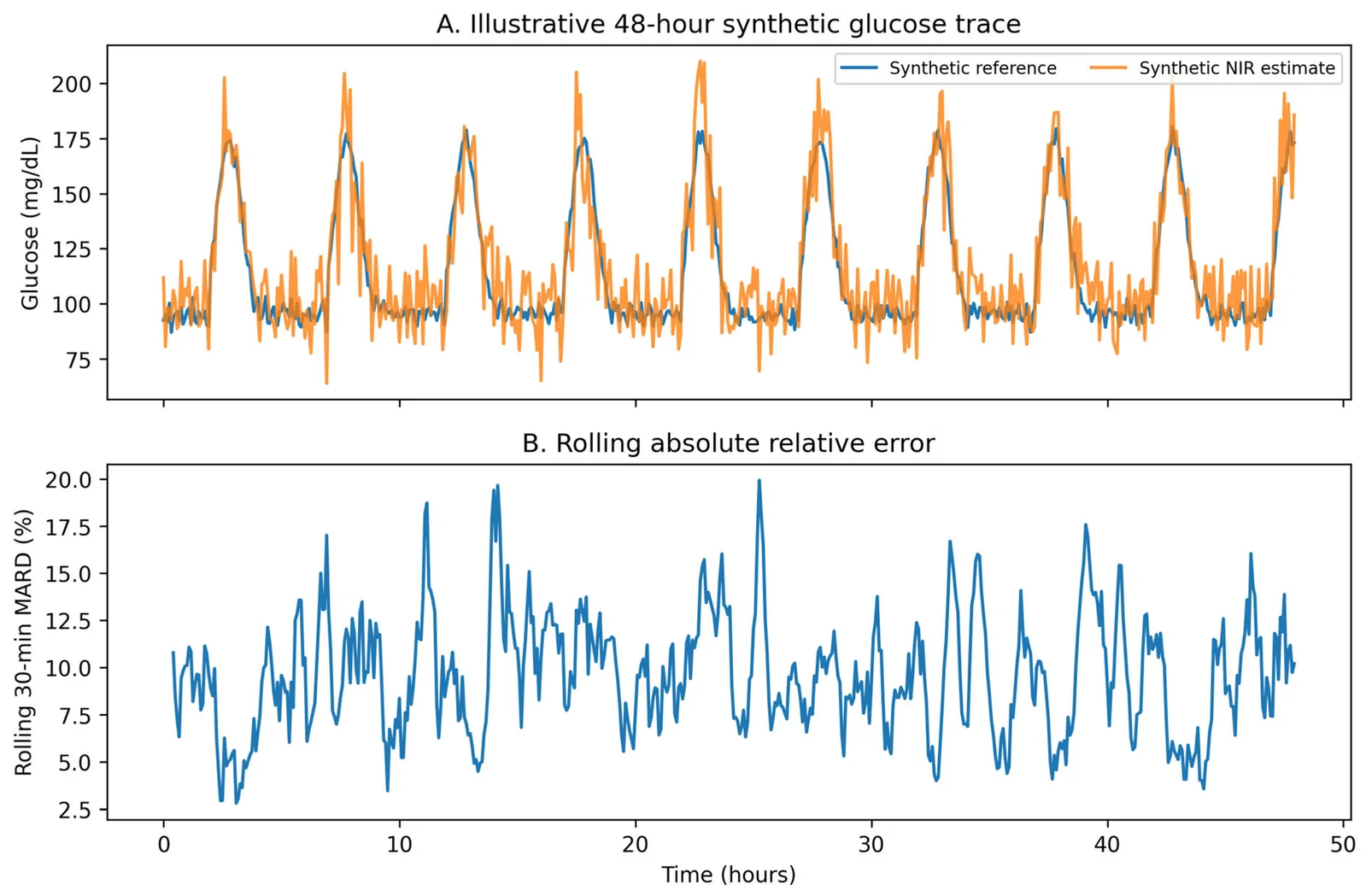
Illustrative 48-h synthetic glucose time series. Panel (**A**) compares the synthetic reference trajectory with the synthetic NIR-estimated trajectory. Panel (**B**) presents the rolling 30-min MARD calculated from the synthetic pairs. These values were generated for illustration and are not observed participant, YSI 2300, or wearable-hardware measurements.

The separate 48-h series is presented only as an illustrative synthetic trace and is not participant-linked validation data. It contains 576 values at 5-min intervals generated from literature-informed meal-response kinetics and noise assumptions. Figure 3 therefore illustrates temporal error behavior without implying actual YSI sampling or prospective wearable monitoring.

The synthetic NIR trajectory follows the broad shape of the generated reference trajectory while exhibiting local deviations introduced by the simulation assumptions. Because meal events, temporal dynamics, and NIR noise were algorithmically generated, visual tracking in Figure 3 cannot be interpreted as evidence of temporal stability, physiological lag correction, or drift mitigation in an implemented sensor.

Demonstrating those properties would require prospectively collected repeated measurements, raw optical signals, participant identifiers, a prespecified calibration-update procedure, and participant-grouped validation over an appropriate wear period.

**Figure 4:**
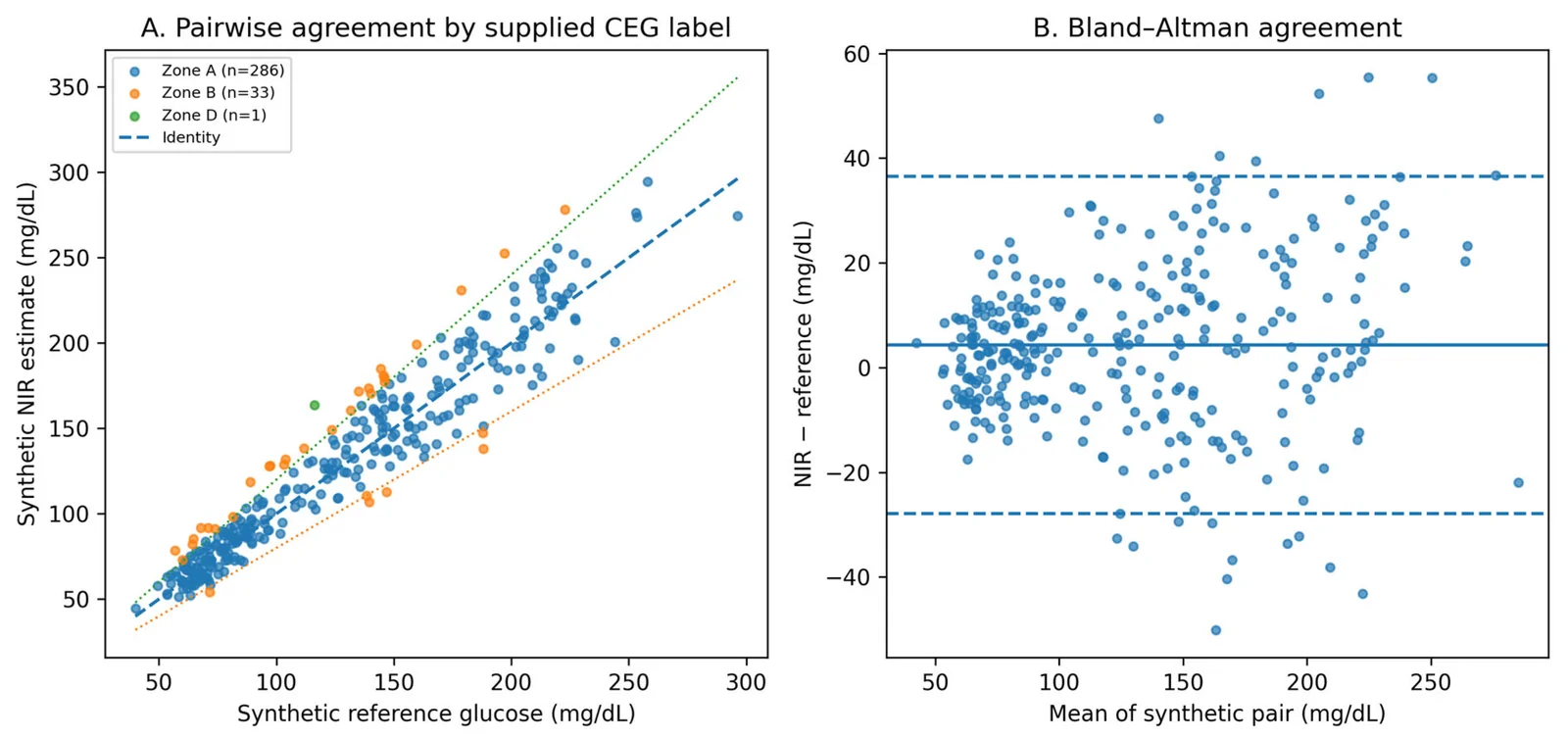
Descriptive agreement of the literature-grounded synthetic glucose pairs (*n* = 320). Panel (**A**) plots the synthetic NIR estimate against the synthetic reference and distinguishes the supplied CEG labels; identity and ±20% guide lines are shown only as visual references and are not a substitute for a complete regulatory assessment. Panel (**B**) shows Bland–Altman differences with a mean bias of 4.32 mg/dL and 95% limits of agreement from −27.86 to 36.50 mg/dL. Source: synthetic supplementary dataset.

Table 4 and Figure 4 summarize the CEG categories stored in the synthetic dataset and the Bland–Altman agreement characteristics. CEG and Bland–Altman outputs are retained as descriptive diagnostics only, they are not used to infer methodological rigor, generalizability, ISO 15197:2013 compliance [21], or FDA iCGM compliance [20].

The corrected CEG distribution comprises 286 records in Zone A (89.4%), 33 in Zone B (10.3%), one in Zone D (0.3%), and none in Zones C or E.

Bland–Altman analysis showed a bias of 4.32 mg/dL with limits of agreement from −27.86 to 36.50 mg/dL. These values quantify agreement within the simulation but are not labeled clinically acceptable because clinical acceptability depends on intended use, prospective device performance, and prespecified regulatory/clinical criteria. Figure 4 visualizes the pairwise relationship, supplied CEG labels, and Bland–Altman differences.

The strong reference–estimate correlation should also be interpreted cautiously. Correlation measures co-variation and does not by itself establish interchangeability, clinical safety, independence from calibration data, or performance on unseen participants. In this simulation, the high r value is partly a consequence of generating estimates around reference values using literature-informed error distributions; it is therefore expected and should not be presented as independent validation evidence.

## 4. Discussion

The revised analysis changes the central interpretation of the study. Rather than validating a wearable NIR glucose monitor, the study demonstrates a literature-grounded simulation that can be used to stress-test analysis choices and formulate prospective calibration hypotheses. The aggregate MARD, correlation, CEG distribution, and Bland–Altman limits reproduce an internally coherent synthetic performance profile, but they cannot establish real-world accuracy because the observations were generated from literature-informed distributions rather than collected from a device in participants.

The confounder analysis is especially important in this reinterpretation because hematocrit had the largest observed positive correlation with MARD (r = 0.090), but its confidence interval included zero, and its Holm-adjusted *p*-value was 0.852. All other candidate-factor correlations were similarly small and non-significant. Accordingly, the data do not support statements that hematocrit, humidity, temperature, pressure, or any other encoded variable is a major confounder in the present simulation. Their inclusion in a future calibration model remains scientifically plausible because optical tissue measurements are affected by absorption, scattering, perfusion, coupling, and motion, but those mechanisms must be evaluated prospectively.

The source study by Cheng et al. [7] describes a fully optical multimodal system combining differential optical absorbance and PPG with temporal modeling. Table 1 makes this distinction explicit. Moreover, although Sensor C has a lower descriptive MARD in the synthetic data, neither ANOVA nor Kruskal–Wallis testing supports a statistically significant difference among the three archetypes.

Gessell and Small [23] reported that updated PLS models based on four constant-analyte augmented samples improved prediction accuracy by approximately 50% relative to models based only on the original calibration data in an in vitro aqueous-sample NIR experiment. The current synthetic workbook does not contain paired fixed-versus-adaptive model predictions or raw spectra, so it cannot reproduce or independently verify that percentage. The literature result is used only to motivate the proposed feedback concept.

A practical adaptive calibration implementation would require an explicit source of reference information. A prospective wearable study could initialize a subject-specific model using reference glucose measurements and subsequently update the model at prespecified intervals when trustworthy reference samples or other calibration anchors are available. The optimal update frequency cannot be inferred from the current simulation and should be chosen prospectively based on observed model drift, sensor stability, and intended use. Importantly, if repeated invasive reference measurements are required for routine updating, the practical advantage of a nominally non-invasive system may be reduced. This trade-off should be evaluated as a design outcome rather than assumed away.

Future validation should therefore separate model development, calibration, and independent assessment. Participant identifiers are essential so that repeated measurements from the same person can be grouped, preventing data leakage between training and validation sets. Raw spectra are needed to evaluate wavelength selection, preprocessing, PLSR or alternative models, and calibration updates. Anatomical site, source–detector geometry, integration time, repeat count, contact pressure, ambient conditions, and motion quality should be recorded prospectively. Skin pigmentation should be characterized using Fitzpatrick phototype and preferably an objective optical or melanin index because it may materially affect transdermal optical measurements [13].

In relation to regulatory interpretation, a favorable MARD or a high percentage of CEG A+B points cannot be equated with FDA iCGM clearance or ISO 15197 compliance. Current 21 CFR §862.1355 requires robust clinical evidence, concentration-specific accuracy proportions with confidence bounds, performance across sensor wear, interference testing, and other special controls [20]. ISO 15197:2013 addresses self-testing systems based on capillary blood samples [21]. For CGM performance-methodology concepts, FDA-recognized CLSI POCT05 is more directly relevant [22]. The present synthetic analysis does not satisfy these clinical or device-validation requirements.

The principal limitation is the synthetic nature of the evidence. No source-study participant-level records, subject identifiers, raw NIR spectra, prospectively acquired YSI measurements, integration-time metadata, repeated-measurement structure, or hardware-level adaptive-calibration outputs are available. The 48-h series is also simulated and not linked to a participant. Source studies used heterogeneous optical architectures, measurement sites, populations, and algorithms; the simulation harmonizes selected benchmark ranges but cannot reproduce all source-specific covariance structures. Finally, skin phototype is unavailable. These limitations restrict the study to methodological and hypothesis-generating conclusions.

## 5. Conclusions

This literature-grounded simulation produced an internally consistent set of descriptive glucose-agreement metrics (overall MARD 10.38%, r = 0.958, and a Bland–Altman bias of 4.32 mg/dL). The CEG distribution was 89.4% in Zone A, 10.3% in Zone B, and 0.3% in Zone D; these categories are descriptive and are not interpreted as ISO or FDA compliance.

None of the eight candidate physiological or environmental variables showed a statistically supported linear association with MARD after multiplicity correction, and the three sensor-archetype MARD distributions did not differ significantly. The candidate variables therefore remain literature-motivated factors for prospective testing rather than demonstrated confounders in the present analysis.

Adaptive calibration is retained as the main forward-looking methodological contribution. The approximately 50% improvement cited in this context derives from prior in vitro work and was not reproduced by the current dataset. A clinically meaningful evaluation will require prospectively acquired raw optical data, participant-grouped validation, skin-phototype characterization, explicit measurement geometry and acquisition settings, and an implemented calibration-update protocol with a prespecified reference strategy.

## Figures and Tables

**Table 1:** Literature-informed sensor archetypes used to parameterize the synthetic dataset.

Archetype	Source	Configuration/Wavelength/Site	Role/Limitation
Sensor A	Rajeswari & Vijayakumar [5]	Reflected-light NIR; 940 nm emitter; 900–1700 nm detector; finger/wrist.	Literature-informed synthetic archetype.
Sensor B	Raghavan et al. [6]	940 nm NIR + PSO-ANN; finger/contact prototype.	Benchmark source; integration time/repeat counts unavailable.
Sensor C	Cheng et al. [7]	Visible–NIR differential absorbance + PPG; multiwavelength; index finger.	Fully optical synthetic archetype; no electrochemical CGM.

**Table 2:** Descriptive statistics of the literature-grounded synthetic glucose dataset by glucose range and sensor archetype (*n* = 320).

Glucose Range/Sensor	*n*	Mean Ref. (mg/dL)	Mean NIR (mg/dL)	MARD (%)	SD MARD	Pearson r	Clarke Zone A (%)
Hypoglycemia (<70)	53	62.3	66.2	11.45	8.53	0.518	90.6
Euglycemia (70–140)	133	98.2	102.5	10.51	8.03	0.876	87.2
Mild hyperglycemia (140–200)	89	164.2	168.0	10.70	7.17	0.572	87.6
Higher hyperglycemia (>200)	45	219.9	225.7	8.11	6.10	0.598	97.8
Overall	320	127.7	132.0	10.38	7.67	0.958	89.4
Sensor A	113	119.6	124.7	10.48	7.55	0.961	90.3
Sensor B	131	127.9	132.3	10.90	8.25	0.956	86.3
Sensor C	76	139.4	142.3	9.34	6.75	0.956	93.4

Note. Ref. = synthetic reference glucose value; NIR = synthetic NIR-estimated glucose value; MARD = mean absolute relative difference; SD = standard deviation; CEG assignments are the categories stored in the synthetic supplementary dataset.

**Table 3:** Pearson associations between MARD and candidate physiological/environmental variables (*n* = 320).

Candidate Variable	Pearson r	95% CI	*p*-Value	Holm-Adjusted *p*	Interpretation
Skin thickness	0.033	[−0.077, 0.142]	0.561	1.000	Not statistically supported
Skin temperature	−0.055	[−0.164, 0.055]	0.324	1.000	Not statistically supported
Relative humidity	0.070	[−0.040, 0.178]	0.211	1.000	Not statistically supported
BMI	−0.082	[−0.190, 0.028]	0.145	1.000	Not statistically supported
Age	0.001	[−0.109, 0.111]	0.985	1.000	Not statistically supported
Hematocrit	0.090	[−0.019, 0.198]	0.107	0.852	Not statistically supported
Motion artifact	0.007	[−0.102, 0.117]	0.897	1.000	Not statistically supported
Sensor pressure	−0.062	[−0.171, 0.048]	0.267	1.000	Not statistically supported

**Table 4:** Descriptive Clarke error grid categories and Bland–Altman agreement for the synthetic dataset (*n* = 320).

Parameter	Value	Interpretation
Clarke Zone A	286 (89.4%)	Supplied CEG category in synthetic dataset
Clarke Zone B	33 (10.3%)	Supplied CEG category in synthetic dataset
Clarke Zone C	0 (0.0%)	No synthetic record assigned
Clarke Zone D	1 (0.3%)	One synthetic record assigned
Clarke Zone E	0 (0.0%)	No synthetic record assigned
Bland–Altman bias	+4.32 mg/dL	Mean synthetic NIR − reference difference
Lower 95% limit of agreement	−27.86 mg/dL	Descriptive agreement limit
Upper 95% limit of agreement	+36.50 mg/dL	Descriptive agreement limit
Pearson r	0.958 (*p* < 0.001)	Linear association within synthetic data
Overall MARD	10.38% (SD 7.67%)	Descriptive synthetic error metric
Regulatory status	Not evaluated	No FDA or ISO compliance claim
Clinical validation	Not performed	Requires prospective human/device data

Note. CEG = Clarke error grid. The CEG categories are those stored in the supplementary synthetic dataset. ISO 15197:2013 applies to capillary blood-glucose self-testing systems [21], and current FDA iCGM special controls require multiple concentration-specific and clinical-validation criteria [19]; therefore, neither CEG A + B percentage nor MARD is treated as evidence of regulatory compliance.

## Data Availability

No new primary human or device measurements were generated. The analyzed records are synthetic and were constructed from literature-reported physiological ranges and performance distributions, with source traceability provided in the Supplementary Workbook. Each synthetic record is explicitly labeled as generated and does not correspond to an identifiable participant or an observed YSI 2300 measurement. The revised Supplementary Workbook contains the data and recalculated inferential summaries used in the manuscript.

## Supplementary Materials

The following supporting information can be downloaded at: https://www.mdpi.com/article/10.3390/s26175443/s1, Supplementary File S1: sensors-26-05443-supplementary_REVISED.xlsx, containing the literature-grounded synthetic NIR glucose dataset, the illustrative 48-h synthetic time series, source traceability, variable dictionary, descriptive statistics, correlation matrix, Clarke Error Grid summary, uncertainty-aware candidate-factor inference, and sensor-archetype summary. The literature sources used to parameterize the synthetic dataset and sensor archetypes are cited in the main manuscript and are identified in the source-traceability fields of Supplementary File S1.

## Abbreviations

The following abbreviations are used in this manuscript:

CEG	Clarke Error Grid
CGM	Continuous Glucose Monitoring
CI	Confidence Interval
FDA	U.S. Food and Drug Administration
iCGM	Integrated Continuous Glucose Monitoring
MARD	Mean Absolute Relative Difference
NIR	Near-Infrared
PLS	Partial Least Squares
PLSR	Partial Least Squares Regression
PPG	Photoplethysmography
SNV	Standard Normal Variate

## Appendix A. Literature-Grounded Synthetic NIR Glucose Dataset with Source Traceability

### NIR_Glucose_Dataset_Sourced

The Supplementary Workbook contains 320 synthetic NIR glucose pairs generated from physiological ranges and performance distributions reported in the cited literature. It includes synthetic reference and NIR-estimated values, MARD, supplied CEG categories, candidate physiological/environmental variables, sensor-archetype labels, source identifiers, URLs, and explicit data-generation notes. The workbook also contains a separate 576-point illustrative 48-h synthetic series. Neither dataset represents measurements acquired by the authors or participant-level observations extracted from the cited studies. Revised sheets provide uncertainty-aware confounder statistics, sensor-archetype summaries, and corrections to source metadata.

## References

[1] Al-Nabulsi J., Abu Owida H., Ma’touq J., Matar S., Al-Aazeh E., Al-Maaiouf A., Bleibel A. (2022). Non-invasive sensing techniques for glucose detection: A review. Bull. Electr. Eng. Inform..

[2] Davison N.B., Gaffney C.J., Kerns J.G. (2022). Recent progress and perspectives on non-invasive glucose sensors. Diabetology.

[3] De Ridder F., Braspenning R., Ordóñez J., Klarenbeek G., Lauwers P., Ledeganck K.J., Delbeke D., De Block C. (2024). Early feasibility study with an implantable near-infrared spectroscopy sensor for glucose, ketones, lactate and ethanol. PLoS ONE.

[4] Irfani M.Z., Koesoema A.P. Continuous and non-invasive blood glucose measurements: A narrative review. Proceedings of the 2023 IEEE ICITACEE.

[5] Rajeswari S.V.K.R., Vijayakumar P. (2024). Development of sensor system and data analytic framework for non-invasive blood glucose prediction. Sci. Rep..

[6] Raghavan D., Suma K., Karki M.V., Sharma N., Rao G. (2023). Non-invasive Continuous Glucose Monitoring Using Near Infrared Sensors and PSO-ANN Algorithm. TechRxiv.

[7] Cheng J., Xie P., Zhao H., Ji Z. (2025). Differential absorbance and PPG-based non-invasive blood glucose measurement using spatiotemporal multimodal fused LSTM model. Sensors.

[8] Dixon J. (2025). Non-Invasive Optical Methods for the Detection of Blood Analytes in the Near Infrared Spectrum. Doctoral Dissertation.

[9] Li T., Wang Q., Lei L., An Y., Guo L., Ren L., Chen X. (2025). Improvement of non-invasive glucose estimation accuracy through multi-wavelength PPG. IEEE J. Biomed. Health Inform..

[10] Vasanthadev S.S., Prince Shanthi A. (2022). Influence of data pre-processing techniques for PLSR model to predict blood glucose by NIR spectroscopy. Opt. Spectrosc..

[11] Fu B., Meng Y., Zhang X., Zhang Z. (2023). A comparison of CNN and PLSR for glucose monitoring using mid-infrared absorption spectroscopy. Open J. Appl. Sci..

[12] Nazha H., Mohsen M., Loutfi A. (2023). Portable infrared-based glucometer reinforced with fuzzy logic. Biosensors.

[13] Dixon C. (2024). Glucose Sensing Using Near Infrared Spectroscopy. Doctoral Dissertation.

[14] Lin B.S., Lin H.Y., Shau Y.C. (2025). Noninvasive blood glucose monitoring system based on deep learning and multiwavelength near-infrared technology. IEEE Trans. Hum.-Mach. Syst..

[15] Agrawal H., Jain P., Joshi A. (2022). Machine learning models for non-invasive glucose measurement: Towards diabetes management in smart healthcare. Health Technol..

[16] González-Viveros N., Castro-Ramos J., Gomez-Gil P., Cerecedo-Núñez H.H., Gutiérrez-Delgado F., Torres-Rasgado E., Pérez-Fuentes R., Flores-Guerrero J.L. (2022). Quantification of glycated hemoglobin and glucose in vivo using Raman spectroscopy and artificial neural networks. Lasers Med. Sci..

[17] Naresh M.U., Peddakrishna S. (2023). Non-invasive near-infrared-based optical glucose detection system for accurate prediction and multi-class classification. Int. J. Exp. Res. Rev..

[18] Yadava D., Singha M., Sharma S., Sharma S., Dubey P.K., Singh S.P. (2022). Dual wavelength based approach with partial least square regression for the prediction of glucose concentration. Indian J. Pure Appl. Phys..

[19] Chen J., Yu K., Zhuang S., Zhang D. (2024). Exploratory insights into prefrontal cortex activity in continuous glucose monitoring: Findings from a portable wearable functional near-infrared spectroscopy system. Front. Neurosci..

[20] Electronic Code of Federal Regulations (2026). 21 CFR § 862.1355—Integrated Continuous Glucose Monitoring System. U.S. Food and Drug Administration. https://www.ecfr.gov/current/title-21/chapter-I/subchapter-H/part-862/subpart-B/section-862.1355.

[21] (2013). In Vitro Diagnostic Test Systems—Requirements for Blood-Glucose Monitoring Systems for Self-Testing in Managing Diabetes Mellitus.

[22] (2021). Recognized Consensus Standard 7-307: CLSI POCT05, 2nd Edition—Performance Metrics for Continuous Interstitial Glucose Monitoring.

[23] Gessell A.J., Small G.W. (2025). Use of constant-analyte samples in updating quantitative near-infrared calibration models for physiological levels of glucose. Anal. Lett..

